# Machine learning methods, applications and economic analysis to predict heart failure hospitalisation risk: a scoping review

**DOI:** 10.1136/bmjopen-2024-093495

**Published:** 2025-06-25

**Authors:** João Abreu, Joana Seringa, Teresa Magalhaes

**Affiliations:** 1 NOVA National School of Public Health, NOVA University Lisbon, Lisbon, Portugal; 2 NOVA National School of Public Health, Public Health Research Centre, Comprehensive Health Research Center (CHRC), REAL, CCAL, NOVA University Lisbon, Lisbon, Portugal

**Keywords:** Heart failure, Machine Learning, Hospitalization, Health economics, CARDIOLOGY

## Abstract

**Abstract:**

**Background:**

Machine Learning (ML) has been transformative in healthcare, enabling more precise diagnostics, personalised treatment regimens and enhanced patient care. In cardiology, ML plays a crucial role in risk prediction and patient stratification, particularly for heart failure (HF), a condition affecting over 64 million people globally and imposing an economic burden of approximately $108 billion annually. ML applications in HF include predictive analytics for risk assessment, identifying patient subgroups with varying prognoses and optimising treatment pathways. By accurately predicting the likelihood of hospitalisation and rehospitalisation, ML tools help tailor interventions, reduce hospital visits, improve patient outcomes and lower healthcare costs.

**Objective:**

To conduct a comprehensive review of existing ML models designed to predict hospitalisation risk in individuals with HF.

**Methods:**

A database search including PubMed, SCOPUS and Web of Science was conducted on 31 March 2024. Studies were selected based on inclusion criteria focusing on ML models predicting hospitalisation risks in adults with HF. The data from 27 studies meeting the criteria were extracted and analysed, with a focus on the predictive performance of the ML models and the presence of economic analysis.

**Results:**

Most studies focused on predicting readmission rather than first-time hospitalisation. All included studies employed supervised ML algorithms, with ensemble-based methods generally yielding the highest predictive performance. For 30-day hospitalisation or readmission risk, Extreme Gradient Boosting (XGBoost) achieved the highest mean area under the curve (AUC) (0.69), followed by Naïve Bayes (0.68) and Deep Unified Networks (0.66). For 90-day risk, the best-performing models were Least Absolute Shrinkage and Selection Operator and Gradient Boosting, both with a mean AUC of 0.75, followed by Random Forest (0.67). When the prediction timeframe was unspecified, Categorical Boosting achieved the highest performance with a mean AUC of 0.88, followed by Generalised Linear Model Net and XGBoost (both 0.79).

Electronic health records were the primary data source across studies; however, few models included patient-reported outcomes or socioeconomic variables.

None of the studies conducted an economic evaluation to assess the cost-effectiveness of these models.

**Conclusions:**

ML holds substantial potential for improving HF care. However, further efforts are needed to enhance the generalisation of models, integrate diverse data sources and evaluate the cost-effectiveness of these technologies.

**STRENGTHS AND LIMITATIONS OF THIS STUDY**

- A key strength lies in identifying ensemble-based models as top performers, providing valuable insights for clinical decision-making and patient management.
- This review’s geographical scope, with an emphasis on studies from the USA, may limit its applicability to other healthcare systems and populations.
- Although machine learning (ML) models show great potential, the absence of economic analyses in the literature limits the ability to assess the cost-effectiveness and practical implementation of these models.
- Despite these limitations, this scoping review serves as an essential foundation for future research that integrates diverse data sources, broadens geographic representation and addresses the economic implications of ML in clinical practice.

## Introduction

 Machine Learning (ML) is revolutionising various sectors, with healthcare being one of the most promising areas of impact.^1^ As a subset of artificial intelligence, ML involves developing algorithms that enable computers to learn from data and make informed decisions.^2^ The recent surge in large-scale data availability and computational advancements has facilitated the adoption of ML in healthcare, offering new avenues for diagnosis, treatment and patient management.^1 3^

ML applications in healthcare are diverse, ranging from enhancing diagnostic imaging to developing personalised medicine approaches.^1 3^ One of the most significant advantages of ML is its capacity to process and analyse complex datasets, such as medical records and imaging data.^1 3^ These capabilities enable the identification of patterns and anomalies that may not be apparent to human clinicians, thus supporting early and accurate diagnoses, tailored treatment plans and the prediction of patient outcomes.^1 3^

A key application of ML in healthcare is in the field of cardiology, particularly in managing heart failure (HF)—a critical public health issue characterised by the heart’s inability to pump blood effectively, leading to symptoms such as fatigue, breathlessness and oedema.^4 5^ Early detection and precise management of HF are crucial for improving patient prognosis and quality of life.^5^

Hospitalisation and rehospitalisation are significant concerns in the management of HF.^5^,^7^ ML models can play a vital role in predicting hospitalisation risks by analysing patient data, including clinical history, lab results and demographic factors.^4^ For instance, predictive analytics can identify patients at high risk of hospitalisation due to worsening symptoms or inadequate response to treatments, enabling healthcare providers to intervene early and potentially prevent hospital admission.^1 8^

Moreover, rehospitalisation is a common issue among HF patients, often due to disease progression or complications.^6 7^ ML can help mitigate this problem by identifying patterns and risk factors associated with rehospitalisation.^4^ For example, algorithms can analyse data from previous hospitalisations, medication adherence and comorbidities to predict which patients are more likely to require readmission.^4^ This information can be used to design targeted interventions, such as closer monitoring, medication adjustments or patient education programmes, thereby reducing the likelihood of rehospitalisation and improving patient outcomes.^4^

This scoping review aims to conduct a comprehensive literature review of existing ML models for predicting hospitalisation risk in individuals with HF. Additionally, it assesses the economic analyses related to implementing these predictive models in clinical practice. By exploring both the clinical and economic dimensions, this review seeks to provide a holistic understanding of the impact of ML-based predictive models in managing HF.

The following specific objectives guided the review:

1. To identify the ML models used for predicting hospitalisation risk in individuals with HF.
2. To determine the data sources and variables used.
3. To identify the predictive performance of these ML models.
4. To summarise the key findings in the literature regarding the application of ML in HF hospitalisation risk prediction.
5. To identify the economic analysis conducted to determine the cost-effectiveness of these models.
6. To provide recommendations for developing and applying ML models to predict HF hospitalisations.

## Methods

### Inclusion and exclusion criteria

Inclusion criteria included studies that focused on ML models used to predict the risk of hospitalisation in HF patients aged 18 years and older. Studies that did not include economic analysis but concentrated on ML models for predicting HF hospitalisations were considered. Exclusion criteria excluded studies whose population did not consist of adult individuals with HF, studies where the cause of hospitalisation was not HF, grey literature (eg, unpublished studies and conference abstracts), systematic reviews and studies in languages other than English. To reach the third specific objective, which was to identify the predictive performance of these ML models, there was a need to exclude articles without performance metric comparison. As the included articles predominantly used the area under the curve (AUC) as a performance metric, articles that did not report performance metrics in terms of AUC were also excluded.

### Search strategy

To identify relevant studies, a database search was conducted on 31 March 2024. The databases included were PubMed, SCOPUS and Web of Science. For data management, including duplicate removal, results were exported to a Word document and later to Rayyan software for title and abstract screening. The search string used was: (“Decompensation” OR “Readmission” OR “Hospitalisation” OR “Worsening”) AND (“Artificial Intelligence” OR “Machine Learning” OR “Deep Learning”) AND (“Heart Failure” OR “Heart Failure, Diastolic” OR “Heart Failure, Systolic” OR “Heart Failure, Chronic” OR “Heart Failure, Acute”) OR (“Economic Analysis” AND (“Decompensation” OR “Readmission” OR “Hospitalisation” OR “Worsening”) AND (“Artificial Intelligence” OR “Machine Learning” OR “Deep Learning”) AND (“Heart Failure” OR “Heart Failure, Diastolic” OR “Heart Failure, Systolic” OR “Heart Failure, Chronic” OR “Heart Failure, Acute”)).

Included articles were reviewed by one reviewer for title and abstract screening. Subsequently, the selected studies were full-text screened by two independent reviewers to determine which studies fit the inclusion criteria. Studies that did not meet the inclusion criteria were eliminated. Two separate reviewers extracted the data, and inter-rater reliability was assessed. Disagreements between the reviewers were resolved through discussion or by consulting a third reviewer. If a study had multiple publications, the latest one was retained.

For data organisation, two researchers collected critical information from relevant studies, including title, publication year, country, focus, data sources, variables, predictive period, ML models, predictive performance, applications and any economic analysis performed. Disagreements were discussed to reach a consensus among the team members.

For the findings report, a descriptive analysis was conducted, consolidating related data segments, extracting deductive codes aligned with the results and assessing inter-rater reliability. The information was organised in a tabular format.

The detailed search strategy is available in online supplemental file 1: Detailed search strategy. The search was conducted in accordance with this previously published protocol^9^.

## Results

**Figure 1:**
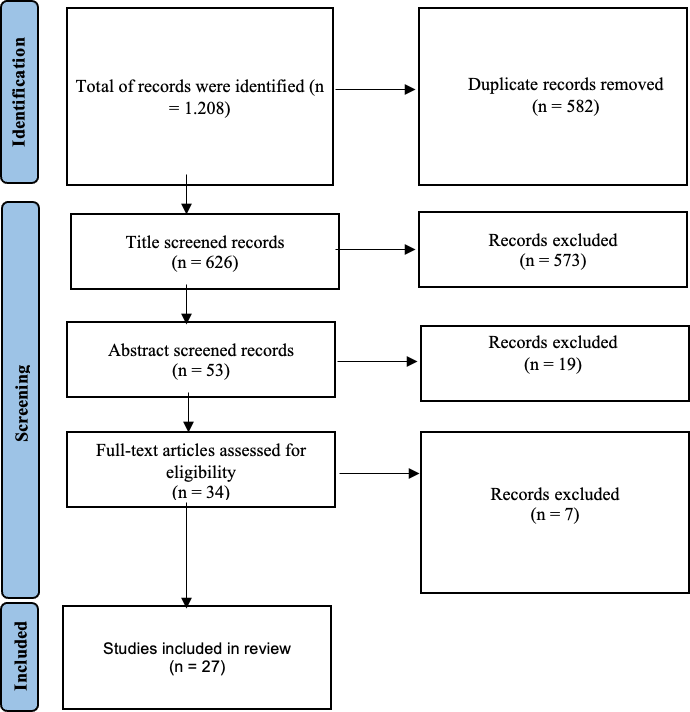
Preferred Reporting Items for Systematic Review and Meta-Analysis Scoping Review flow chart depicting the search strategy.

In the literature search, 1208 references were initially identified. After eliminating duplicates, 626 unique references remained. Subsequent title screening reduced this number to 53. Following abstract screening, 34 articles were selected for full-text review. Ultimately, 27 articles met the criteria and were included in the final review. The full selection process is illustrated in figure 1 (Preferred Reporting Items for Systematic Review and Meta-Analysis extension for Scoping Reviews flow chart).

### Description

The characteristics of the full-text articles included in the study are detailed in online supplemental table 1, (summary of articles investigating ML approaches to predict HF hospitalisation risk).

These articles were published between 2016 and March 2024. The included articles mentioned ML methods tested in various countries: USA (n=11), China (n=3), Spain (n=3), Iran (n=3), Canada (n=2), Japan (n=1), Italy (n=1), Netherlands (n=1), Sweden (n=1), Australia (n=1), Brazil (n=1), Argentina (n=1) and Rwanda (n=1). Among these articles, 20 focused on ML methods to predict HF readmission risk, and 7 focused on ML methods to predict HF hospitalisation risk. The articles also mentioned medical records as the data source for these ML methods (n=24), and both medical records and patient surveys (n=3). The ML methods used different types of variables: clinical (n=6), clinical and demographic (n=14), and clinical, demographic and healthcare utilisation (n=7).

The ML methods encompassed various predictive ML models with different predictive periods and predictive performance metrics, as detailed in online supplemental table 1. Within these articles, the most mentioned predictive periods were 30-day (n=9), non-specified (n=5) and 90-day (n=3).

The articles reviewed identified the top-performing models for predicting 30-day HF hospitalisation/readmission risk. The highest performance was achieved by the Extreme Gradient Boosting (XGBoost) (ensemble-based) model with a mean AUC of 0.69, followed by the Naïve Bayes Classifier (Bayesian-based) with a mean AUC of 0.68 and the Deep Learning—Deep Unified Networks (neural network-based) with a mean AUC of 0.66. When considering models mentioned in at least three articles for the 30-day prediction, the leading models were XGBoost with a mean AUC of 0.69, Support Vector Machine (instance-based) with a mean AUC of 0.63, Deep Learning—Deep Unified Networks with a mean AUC of 0.63 and Logistic Regression (regularisation-based) with a mean AUC of 0.63.

For predicting 90-day HF hospitalisation/readmission risk, the best results were achieved by the Least Absolute Shrinkage and Selection Operator (regularisation-based) model with a mean AUC of 0.75, followed by the Gradient Boosting (ensemble-based) with a mean AUC of 0.75 and the Random Forest (tree-based) with a mean AUC of 0.67. When considering models mentioned in at least three articles for the 90-day prediction, the leading models were Random Forest with a mean AUC of 0.67, and Logistic Regression (regularisation-based) with a mean AUC of 0.65.

The review also identified top-performing models for predicting HF hospitalisation or readmission risk over a non-specific period. The highest performance was achieved by the Categorical Boosting (ensemble-based) model with a mean AUC of 0.88, followed by the Generalised Linear Model Net (regularisation-based) with a mean AUC of 0.79 and the XGBoost with a mean AUC of 0.79. When focusing on models mentioned in at least three articles for non-specific period predictions, the leading models were Gradient Boosting with a mean AUC of 0.74, Random Forest with a mean AUC of 0.70 and Support Vector Machine with a mean AUC of 0.68.

Key findings in the literature regarding the application of ML in HF hospitalisation risk prediction are summarised in online supplemental table 1. They were organised into four categories: clinical decision support and personalised care, data integration and advanced modelling, monitoring and real-time analytics, and resource planning and management.

#### Clinical decision support and personalised care

Clinical decision support and personalised care (n=26) encompasses the use of ML to enhance clinical decision-making and provide personalised care to HF patients. ML models analyse patient data to predict outcomes, recommend treatments and assist healthcare providers in making evidence-based decisions.^10^ This includes predictive analytics for forecasting patient outcomes such as the likelihood of hospitalisation or readmission, which enables timely interventions.^10^ Additionally, personalised treatment plans are tailored based on individual patient profiles, improving the effectiveness of care.^10^ Risk stratification is another crucial aspect, where high-risk patients who may need more intensive monitoring or interventions are identified.^11^

#### Data integration and advanced modelling

Data integration and advanced modelling (n=16) involves the integration of diverse data sources and the application of advanced ML models to improve the accuracy and robustness of predictions. This category focuses on combining clinical, demographic and other relevant data to provide a comprehensive view of patient health. Multimodal data integration is essential, combining information from electronic health records (EHRs), patient surveys, wearable devices and other sources.^12^

#### Monitoring and real-time analytics

Monitoring and real-time analytics (n=14) highlight the use of ML for continuous monitoring and real-time analysis of patient data. The primary goal is to detect early signs of deterioration and provide timely alerts to healthcare providers, thereby preventing hospitalisations.^13^ Real-time monitoring employs sensors, wearable devices and remote monitoring systems to collect and analyse patient data continuously.^13^ Early warning systems are developed to detect early signs of HF exacerbation and alert healthcare providers.^13^ This real-time feedback mechanism also empowers patients with self-management tools, enabling them to actively participate in their own care.^13^

#### Resource planning and management

Resource planning and management (n=15) focuses on the application of ML to optimise resource allocation and management in healthcare settings. By predicting patient needs and hospital utilisation, ML helps in efficient resource planning and reducing healthcare costs.^14^ Capacity planning involves predicting hospital admissions and bed occupancy to ensure adequate resource availability.^14^ Forecasting staffing needs based on predicted patient influx ensures optimal staffing levels, thereby enhancing operational efficiency.^14^ Furthermore, cost management identifies cost-saving opportunities by predicting high-cost patients and optimising care pathways, contributing to more efficient healthcare delivery.^14^

Economic analyses conducted to determine the cost-effectiveness of the application of ML models were notably absent in the reviewed literature.

## Discussion

This article comprehensively reviews the application of ML models for predicting HF hospitalisation and readmission risk in different healthcare contexts.

A notable limitation of this review is the geographical concentration of the included studies, with approximately 41% originating from the USA. This may reflect the country’s strong research infrastructure and access to large healthcare datasets.^15 16^ However, it also raises concerns about the generalisability of findings, as healthcare systems, practices and patient demographics vary significantly across regions. Broader international representation is needed to ensure the global applicability of ML models in HF care.^17^

A notable finding from this review is the greater emphasis placed on predicting HF readmission risk compared with initial hospitalisation risk. One key reason for this focus could be the limited availability of data before a patient’s first hospitalisation.^18 19^ Primary care records are frequently maintained in systems separate from those used by hospitals, which presents challenges to fully integrate patient data and results in less comprehensive datasets for ML models.^20^ Future research should explore innovative data integration approaches that link primary care, specialty care and hospital data. Moreover, incorporating emerging data sources, such as wearable devices and home monitoring systems, could provide earlier insights into HF progression, ultimately improving the predictive accuracy of models for first-time hospitalisations.^21^ In contrast, after an initial hospitalisation, more detailed data are collected, providing a richer foundation for predictive modelling.^18 19^ Furthermore, the emphasis on readmission risk may be influenced by policy changes and reimbursement practices, such as penalties imposed on hospitals with high readmission rates through initiatives like the Hospital Readmissions Reduction Programme.^22^,^24^

The review also highlights that the majority of these ML methods relied on medical records, while a smaller subset of methods used both medical records and patient surveys. This reliance on medical records suggests that EHRs continue to be the primary data source for ML in healthcare, likely due to their structured nature and widespread availability.^18 19 25^ However, future research should focus on incorporating patient-reported outcomes (PROs), such as surveys capturing symptoms and quality of life, into these models.^26^ PROs provide valuable insights that are often missing from clinical records, offering a more comprehensive view of a patient’s health status.^26^ Integrating these multimodal data sources, including medical records, may enhance the accuracy and applicability of ML models by accounting for real-world patient experiences.

Another key finding is the relevance of demographic variables within the models reviewed. The integration of demographic factors, particularly socioeconomic status, is closely tied to treatment adherence and a patient’s ability to manage the chronic condition of HF effectively.^17 27 28^ Patients from lower socioeconomic backgrounds often encounter obstacles such as limited access to healthcare services, lower health literacy and financial constraints, all of which can significantly hinder their ability to manage the disease over time.^17 27 28^ Future research should continue to investigate the impact of these demographic factors while also developing models that address disparities and promote equitable care across different socioeconomic groups.

Another notable observation from the studies reviewed is the consistent use of supervised learning models, with ensemble-based models demonstrating slightly superior performance. This preference for supervised learning models can be attributed to the nature of HF hospitalisation risk prediction, which involves clearly labelled training data.^29^,^31^ These models are particularly well-suited for tasks where the relationship between input features—such as clinical, demographic and healthcare utilisation variables—and output labels, like hospitalisation, needs to be learnt.^29^,^31^

Ensemble-based models performed slightly better, perhaps due to their ability to combine the strengths of multiple weaker models, resulting in more robust and accurate predictions.^29^,^31^ By averaging the predictions of various models or weighting them appropriately, ensemble methods help to reduce variance and avoid overfitting.^29^,^31^ This is particularly important for capturing complex patterns in healthcare data that might be missed by a single model.^32^,^34^ Ensemble methods are especially valuable when dealing with high-dimensional datasets, as they can effectively manage numerous variables, identify the most relevant features and minimise noise.^32^,^34^ Based on the studies reviewed, ensemble-based models appear to be the most promising ML approach for predicting HF hospitalisation and readmission. Their consistent performance across diverse datasets, ability to manage high-dimensional and imbalanced data, and robustness against overfitting make them particularly well-suited for clinical applications in this domain.^32^,^34^

The complexity of healthcare data, with its non-linear interactions between clinical, demographic and healthcare utilisation variables, makes ensemble methods particularly effective.^32^,^34^ Their ability to aggregate diverse decision boundaries enables them to capture nuanced relationships within the data that could be crucial for predicting outcomes like HF hospitalisation.^32^,^34^ Additionally, ensemble models are adept at handling unbalanced datasets, which are common in healthcare settings where hospitalisation events are relatively infrequent.^32^,^34^ By iteratively refining their predictions, these models improve sensitivity to minority classes, such as patients at high risk for hospitalisation or readmission, while maintaining overall predictive accuracy.^32^,^34^ This ability to focus on high-risk individuals makes ensemble models especially valuable in healthcare, where timely and accurate identification of these patients can lead to more effective interventions and better patient outcomes.^32^,^34^

The review underscores the transformative potential of ML in predicting HF hospitalisation across several key areas. Clinical decision support and personalised care emerged as particularly impactful, demonstrating how ML enhances risk stratification and tailors treatment plans, improving patient outcomes and enabling more effective interventions. Data integration and advanced modelling highlight the value of combining diverse data sources, such as EHRs and patient surveys, to develop more accurate and robust predictive models. Monitoring and real-time analytics showcase the promise of continuous patient monitoring and early detection of deterioration, which can help prevent unnecessary hospitalisations and support patient self-management. Finally, resource planning and management illustrate how ML can optimise resource allocation and reduce costs by predicting patient needs and hospital utilisation. However, a significant gap identified in the literature is the absence of comprehensive economic analyses to assess the cost-effectiveness of ML model applications. Future research must prioritise these economic evaluations to justify the widespread implementation of ML in clinical settings, particularly in resource-constrained environments. These analyses should consider both the direct costs of implementation and long-term savings from improved patient outcomes, reduced readmissions and optimised resource utilisation, ensuring that adopting these models is economically viable and beneficial to healthcare systems.

## Conclusions

This scoping review synthesised the existing evidence on ML methods, their applications and the current lack of economic analysis in predicting HF hospitalisation risk. The review highlights that ML has shown considerable promise in supporting HF management, particularly in predicting both hospitalisation and readmission risks. Ensemble-based models emerged as top performers due to their ability to handle complex, multimodal healthcare data. These models enhance clinical decision support by enabling risk stratification, personalising care plans and informing early interventions that can improve patient outcomes and reduce hospitalisations.

However, a significant geographical concentration in the USA and an over-reliance on medical records emphasise the need for broader, more inclusive research that incorporates diverse populations and richer data sources, such as PROs. Furthermore, the dominance of studies focusing on readmission risk reflects data limitations prior to initial hospitalisations, suggesting the need for innovative data integration approaches.

Despite these advancements, a major gap identified in the literature is the absence of comprehensive economic analyses. To date, no study has evaluated the cost-effectiveness of implementing ML models in clinical practice. The literature reveals a gap in economic evaluations of ML models for HF hospitalisation prediction. This gap suggests an opportunity for future research to explore the cost-effectiveness and practical implementation considerations of these models in diverse healthcare settings. By doing so, the adoption of ML models can be more effectively justified and tailored to meet the needs of diverse healthcare systems, ensuring both clinical and economic viability.

In conclusion, while ML offers substantial potential for improving HF care, ensemble-based models appear to be the most effective due to their consistent performance, capacity to manage complex and high-dimensional healthcare data, and ability to capture non-linear interactions among clinical, demographic and utilisation variables. Their robustness and adaptability make them especially suitable for real-world healthcare settings, where data are often heterogeneous and imbalanced. Future research should aim to expand the generalisability of these models, incorporate diverse data sources and conduct robust economic evaluations. Addressing these challenges will help translate the promising results of ML into widespread clinical practice, ultimately reducing hospitalisations and improving patient outcomes on a global scale.

## Supplementary material

10.1136/bmjopen-2024-093495 online supplemental file 1

10.1136/bmjopen-2024-093495 online supplemental file 2

## Data Availability

Data are available in a public, open access repository.
