# Supplementary material for: Machine learning methods, applications and economic analysis to predict heart failure hospitalisation risk: a scoping review
Source: BMJ Open. 2025 Jun 25;15(6):e093495. doi: 10.1136/bmjopen-2024-093495 (PMC12198813; doi:10.1136/bmjopen-2024-093495)
Supplement: online supplemental file 1 [file bmjopen-15-6-s001.docx]

**Supplementary File I – Detailed Search Strategy**

**This search strategy was implemented according to the published protocol.**

- Protocol Reference: BMJ Open (DOI: 10.1136/bmjopen-2023-083188);
- Search Date: March 31, 2024;
- Databases Searched: PubMed, Scopus, Web of Science.

**Search String in all databases:**

("Decompensation" OR "Readmission" OR "Hospitalisation" OR "Worsening") AND ("Artificial Intelligence" OR "Machine Learning" OR "Deep Learning") AND ("Heart Failure" OR "Heart Failure, Diastolic" OR "Heart Failure, Systolic" OR "Heart Failure, Chronic" OR "Heart Failure, Acute") OR ("Economic Analysis" AND ("Decompensation" OR "Readmission" OR "Hospitalisation" OR "Worsening") AND ("Artificial Intelligence" OR "Machine Learning" OR "Deep Learning") AND ("Heart Failure" OR "Heart Failure, Diastolic" OR "Heart Failure, Systolic" OR "Heart Failure, Chronic" OR "Heart Failure, Acute")).

**Protocol Specifications Met:**

- Three major databases searched (PubMed, SCOPUS, Web of Science);
- Comprehensive search terms covering ML/AI and heart failure;
- Economic analysis terms included as specified;
- No date restrictions applied;
- English language restriction applied.
- Adult population focus maintained.

**Adaptations from Protocol:**

No adaptations were made from the published protocol.

**Search Results Summary:**

| **Database** | **Initial Results** | **Final Included Articles** |
| --- | --- | --- |
| PubMed | 186 | 13 |
| Scopus | 665 | 7 |
| Web of Science | 357 | 7 |
| 1.208 | 27 |  |
