# Supplementary material for: Machine learning methods, applications and economic analysis to predict heart failure hospitalisation risk: a scoping review
Source: BMJ Open. 2025 Jun 25;15(6):e093495. doi: 10.1136/bmjopen-2024-093495 (PMC12198813; doi:10.1136/bmjopen-2024-093495)
Supplement: online supplemental file 2 [file bmjopen-15-6-s002.docx]

**Supplementary File II: Summary of articles investigating ML approaches to predict heart failure hospitalisation risk**

Table I – Summary of articles investigating ML approaches to predict heart failure hospitalisation risk

| **Title** | **Publication year** | **Country** | **Focus** | **Data Sources** | **Variables** | **Predictive Period** | **Machine Learning Models** | **Machine Learning Models - Predictive Performance (AUC)** | **Applications of Machine Learning in Heart Failure Hospitalisation Risk Prediction** | **Economic Analysis** |
| --- | --- | --- | --- | --- | --- | --- | --- | --- | --- | --- |
| A machine learning model to predict heart failure readmission: toward optimal feature set^(15)^ | 2024 | United States of America | Readmission | Medical Records | Clinical and Demographic | 30-day | Random Forest Logistic Regression | Random Forest 0.61 Logistic Regression 0.60 | Clinical Decision Support and Personalized Care  Data Integration and Advanced Modelling  Monitoring and Real-Time Analytics  Resource Planning and Management | None |
| A machine learning model to predict the risk of 30-day readmissions in patients with heart failure: a retrospective analysis of electronic medical records data^(16)^ | 2018 | United States of America | Readmission | Medical Records | Clinical, Demographic and Healthcare Utilization | 30-day | Deep Learning - Deep Unified Networks Deep Learning - Maxout Networks Logistic Regression Gradient Boosting | Deep Learning - Deep Unified Networks 0.71 (95% CI: 0.69-0.72) Deep Learning - Maxout Networks 0.70 (95% CI: 0.68-0.71) Logistic Regression 0.66 (95% CI: 0.65-0.68) Gradient Boosting 0.65 (95% CI: 0.64-0.66) | Clinical Decision Support and Personalized Care  Resource Planning and Management | None |
| Analysis of Machine Learning Techniques for Heart Failure Readmissions^(17)^ | 2016 | United States of America | Readmission | Medical Records and Patient Survey | Clinical and Demographic | 30-day | Boosting Random Forest | Boosting 0.68 (95% CI: 0.66-0.69) Random Forest 0.67 (95% CI: 0.66-0.68) | Clinical Decision Support and Personalized Care  Resource Planning and Management | None |
| Comparing Machine Learning Classifiers for Predicting Hospital Readmission of Heart Failure Patients in Rwanda^(18)^ | 2023 | Rwanda | Readmission | Medical Records | Clinical and Demographic | 20 days after discharge | Random Forest Deep Learning - Multi-Layer Perceptrons Support Vector Machine - Linear Support Vector Machine - Radial Basis Function Kernel K-Nearest Neighbors Logistic Regression Decision Trees | Random Forest 0.94 Deep Learning - Multi-Layer Perceptrons 0.88 Support Vector Machine - Linear 0.88 Support Vector Machine - Radial Basis Function Kernel 0.88 K-Nearest Neighbors 0.88 Logistic Regression 0.81 Decision Trees 0.57 | Clinical Decision Support and Personalized Care  Data Integration and Advanced Modelling  Resource Planning and Management | None |
| Comparison of Machine Learning Algorithms for Predicting Hospital Readmissions and Worsening Heart Failure Events in Patients with Heart Failure with Reduced Ejection Fraction: Modelling Study^(19)^ | 2023 | United States of America | Readmission | Medical Records | Clinical, Demographic and Healthcare Utilization | 30-day, 90-day and 365-day | Extreme Gradient Boosting Random Forest Logistic Regression Deep Learning - Multi-Layer Perceptrons | Extreme Gradient Boosting 0.60, 0.63 and 0.65 Random Forest 0.58, 0.63 and 0.62 Logistic Regression 0.58, 0.62 and 0.61 Deep Learning - Multi-Layer Perceptrons 0.58, 0.61 and 0.62 | Data Integration and Advanced Modelling | None |
| Comparison of Machine Learning Methods with Traditional Models for Use of Administrative Claims with Electronic Medical Records to Predict Heart Failure Outcomes^(20)^ | 2020 | United States of America | Readmission | Medical Records | Clinical and Demographic | 365-day | Gradient Boosting Random Forest Least Absolute Shrinkage and Selection Operator (LASSO) Combined Classification and Regression Trees | Gradient Boosting 0.78 (95% CI: 0.75-0.80) Random Forest 0.76 (95% CI: 0.75-0.79) LASSO 0.76 (95% CI: 0.74-0.79)  Combined Classification and Regression Trees 0.74 (95% CI: 0.71-0.77) | Clinical Decision Support and Personalized Care  Monitoring and Real-Time Analytics | None |
| Comparison of machine learning techniques for prediction of hospitalisation in heart failure patients^(21)^ | 2019 | Italy | Hospitalisation | Medical Records | Clinical | Non specified | Generalized Linear Model Net Random Forest Support Vector Machine Deep Learning - Neural Networks Adaptive Boosting Logit Boost Logistic Regression Combined Classification and Regression Trees | Generalized Linear Model Net 0.80 Random Forest 0.70 Support Vector Machine 0.70  Deep Learning - Neural Networks 0.68 Adaptive Boosting 0.65 Logit Boost 0.65 Logistic Regression 0.65  Combined Classification and Regression Trees 0.59 | Clinical Decision Support and Personalized Care  Monitoring and Real-Time Analytics | None |
| Endpoint prediction of heart failure using electronic health records^(22)^ | 2020 | China | Readmission | Medical Records | Clinical and Demographic | 90-day to 730-day | Deep Learning - Deep Active Learning with Ensemble Pruning Deep Learning - Multi-Task Learning with Ensemble Pruning Deep Learning - Gated Recurrent Unit Deep Learning - Reverse Time Attention Model Support Vector Machine Random Forest Deep Learning - Long Short-Term Memory Deep Learning - Denoising Autoencoder Deep Learning - Logistic Regression Extreme Gradient Boosting | Deep Learning - Deep Active Learning with Ensemble Pruning 0.66 (95% CI: 0.65-0.667) Deep Learning - Multi-Task Learning with Ensemble Pruning 0.66 (95% CI: 0.65-0.66) Deep Learning - Gated Recurrent Unit 0.64 (95% CI: 0.63-0.65) Deep Learning - Reverse Time Attention Model 0.64 (95% CI: 0.63-0.65)  Support Vector Machine 0.64 (95% CI: 0.63-0.65) Random Forest 0.64 (95% CI: 0.63-0.65) Deep Learning - Long Short Term Memory 0.63 (95% CI: 0.62-0.64) Deep Learning - Denoising Autoencoder 0.63 (95% CI: 0.62-0.64) Deep Learning - Logistic Regression 0.62 (95% CI: 0.61-0.63) Extreme Gradient Boosting 0.62 (95% CI: 0.61-0.63) | Clinical Decision Support and Personalized Care  Monitoring and Real-Time Analytics | None |
| Heart Failure Emergency Readmission Prediction Using Stacking Machine Learning Model^(23)^ | 2023 | China | Readmission | Medical Records | Clinical and Demographic | Non specified | Extreme Gradient Boosting Categorical Boosting Adaptive Boosting Gradient Boosting | Extreme Gradient Boosting 0.88 Categorical Boosting 0.88 Adaptive Boosting 0.87 Gradient Boosting 0.86 | Clinical Decision Support and Personalized Care  Monitoring and Real-Time Analytics | None |
| HR-BGCN: Predicting readmission for heart failure from electronic health records^(24)^ | 2024 | United States of America | Readmission | Medical Records | Clinical | Until 30 days and after 30 days | Deep Learning - Bidirectional Graph Convolutional Network | Deep Learning - Bidirectional Graph Convolutional Network 0.94 and 0.95 | Clinical Decision Support and Personalized Care  Data Integration and Advanced Modelling  Monitoring and Real-Time Analytics  Resource Planning and Management | None |
| Machine learning based readmission and mortality prediction in heart failure patients^(25)^ | 2023 | Iran | Readmission | Medical Records | Clinical, Demographic and Healthcare Utilization | 30-day and 90-day | Support Vector Machine Logistic Regression Extreme Gradient Boosting Deep Learning - Multi-Layer Perceptrons Random Forest K-Nearest Neighbors | Support Vector Machine 0.73 and 0.59 Logistic Regression 0.71 and 0.59 Extreme Gradient Boosting 0.71 and 0.58 Deep Learning - Multi-Layer Perceptrons 0.67 and 0.58 Random Forest 0.66 and 0.61 K-Nearest Neighbors 0.59 and 0.60 | Clinical Decision Support and Personalized Care  Data Integration and Advanced Modelling  Monitoring and Real-Time Analytics  Resource Planning and Management | None |
| Machine Learning Patient-Specific Prediction of Heart Failure Hospitalisation Using Cardiac MRI-Based Phenotype and Electronic Health Information^(26)^ | 2022 | Canada | Hospitalisation | Medical Records | Clinical and Demographic | 90-day, 365-day and 730-day (2-year) | Random Forest | Random Forest 0.87, 0.83 and 0.80 | Clinical Decision Support and Personalized Care  Data Integration and Advanced Modelling  Monitoring and Real-Time Analytics | None |
| Machine Learning Prediction of Mortality and Hospitalisation in Heart Failure with Preserved Ejection Fraction^(27)^ | 2020 | United States of America, Canada, Brazil and Argentina | Hospitalisation | Medical Records | Clinical | Until 1095 days (3-year) | Random Forest Gradient Descent Boosting Combined Logistic Regression and LASSO Logistic Regression Support Vector Machine - Radial Basis Function Support Vector Machine - Linear Kernel | Random Forest 0.76 (95% CI: 0.71-0.81) Gradient Descent Boosting 0.73 (95% CI: 0.69-0.77) Combined Logistic Regression and LASSO 0.73 (95% CI: 0.67-0.79) Logistic Regression 0.73 (95% CI: 0.66-0.80) Support Vector Machine - Radial Basis Function 0.72 (95% CI: 0.65-0.79) Support Vector Machine - Linear Kernel 0.72 (95% CI: 0.63-0.81) | Clinical Decision Support and Personalized Care  Data Integration and Advanced Modelling  Monitoring and Real-Time Analytics  Resource Planning and Management | None |
| Machine learning-based prediction of heart failure readmission or death: implications of choosing the right model and the right metrics^(28)^ | 2019 | Australia | Readmission | Medical Records | Clinical, Demographic and Healthcare Utilization | 30-day | Deep Learning - Multi-Layer Perceptions Logistic Regression Weighted Random Forest  Weighted Vector Machine Weighted Decision Trees Support Vector Machine Decision Trees Random Forest | Deep Learning - Multi-Layer Perceptions 0.63 Logistic Regression 0.58 Weighted Random Forest 0.55  Weighted Vector Machine 0.54 Weighted Decision Trees 0.53 Support Vector Machine 0.53 Decision Trees 0.52 Random Forest 0.50 | Clinical Decision Support and Personalized Care  Data Integration and Advanced Modelling  Monitoring and Real-Time Analytics | None |
| Predicting 30-day readmission in heart failure using machine learning techniques^(29)^ | 2018 | Spain | Readmission | Medical Records | Clinical | 30-day | Naïve Bayes Classifier Support Vector Machine Combined Decision Trees and Naïve Bayes Classifier Weighted Naïve Bayes Classifier | Naïve Bayes Classifier 0.62 Support Vector Machine 0.62 Combined Decision Trees and Naïve Bayes Classifier 0.61 Weighted Naïve Bayes Classifier 0.58 | Clinical Decision Support and Personalized Care  Data Integration and Advanced Modelling | None |
| Predicting 90-day acute heart failure readmission and death using machine learning-supported decision analysis^(30)^ | 2020 | United States of America | Readmission | Medical Records | Clinical and Demographic | 90-day | Combined Logistic Regression and LASSO Gradient Boosting LASSO Logistic Regression Support Vector Machine Random Forest | Combined Logistic Regression and LASSO 0.76 (95% CI: 0.75-0.77) Gradient Boosting 0.75 (95% CI: 0.74-0.76) LASSO 0.75 (95% CI: 0.74-0.76) Logistic Regression 0.74 (95% CI: 0.73-0.756) Support Vector Machine 0.72 (95% CI: 0.70-0.73) Random Forest 0.57 (95% CI: 0.55-0.59) | Clinical Decision Support and Personalized Care  Resource Planning and Management | None |
| Predicting hospital readmission in heart failure patients in Iran: A comparison of various machine learning methods^(31)^ | 2021 | Iran | Readmission | Medical Records | Clinical and Demographic | Non specified | Support Vector Machine Least-Squares Support Vector Machine Bagging Adaptive Boosting Random Forest Naïve Bayes Classifier | AUC not available (only sensitivity, specificity, precision, negative predictive value and accuracy) | Clinical Decision Support and Personalized Care  Data Integration and Advanced Modelling  Monitoring and Real-Time Analytics  Resource Planning and Management | None |
| Predicting Six-Month Re-Admission Risk in Heart Failure Patients Using Multiple Machine Learning Methods: A Study Based on the Chinese Heart Failure Population Database^(32)^ | 2023 | China | Readmission | Medical Records | Clinical, Demographic and Healthcare Utilization | 180-day | Logistic Regression Naïve Bayes Classifier Regression Trees Random Forest Extreme Gradient Boosting | Logistic Regression 0.63 (95% CI: 0.60–0.65) Naïve Bayes Classifier 0.59 (95% CI: 0.55–0.62) Regression Trees 0.59 (95% CI: 0.49–0.70) Random Forest 0.58 (95% CI: 0.29-0.86) Extreme Gradient Boosting 0.55 (95% CI: 0.39–0.70) | Clinical Decision Support and Personalized Care  Data Integration and Advanced Modelling  Monitoring and Real-Time Analytics  Resource Planning and Management | None |
| Predicting the risk of mortality and rehospitalisation in heart failure patients: A retrospective cohort study by machine learning approach^(33)^ | 2024 | Iran | Readmission | Medical Records | Clinical and Demographic | Until 1825 days (5-year) | Gaussian Naive Bayes Linear Discriminant Analysis Categorical Boosting Logistic Regression Random Forest Extreme Gradient Boosting Gradient Boosting Support Vector Machine K-Nearest Neighbors Decision Trees | Gaussian Naive Bayes 0.75 (95% CI: 0.70–0.80) Linear Discriminant Analysis 0.74 (95% CI: 0.69–0.80) Categorical Boosting 0.74 (95% CI: 0.68–0.79) Logistic Regression 0.73 (95% CI: 0.68–0.79) Random Forest 0.73 (95% CI: 0.67–0.78)  Extreme Gradient Boosting 0.71 (95% CI: 0.66–0.77)  Gradient Boosting 0.67 (95% CI: 0.61–0.73) Support Vector Machine 0.63 (95% CI: 0.57–0.70) K-Nearest Neighbors 0.59 (95% CI: 0.52–0.66) Decision Trees 0.54 (95% CI: 0.47–0.61) | Clinical Decision Support and Personalized Care  Data Integration and Advanced Modelling  Monitoring and Real-Time Analytics  Resource Planning and Management | None |
| Prediction and Analysis of Heart Failure Decompensation Events Based on Telemonitored Data and Artificial Intelligence Methods^(34)^ | 2023 | Spain | Hospitalisation | Medical Records and Patient Survey | Demographic and Clinical | Non specified | Extreme Gradient Boosting Adaptive Boosting Gradient Boosting Logistic Regression Support Vector Machine Bagging Random Forest Extra Trees | Extreme Gradient Boosting 0.69  Adaptive Boosting 0.69 Gradient Boosting 0.68 Logistic Regression 0.68 Support Vector Machine 0.67 Bagging 0.66 Random Forest 0.66 Extra Trees 0.67 | Clinical Decision Support and Personalized Care  Data Integration and Advanced Modelling | None |
| Prediction of heart failure decompensations using artificial intelligence techniques^(35)^ | 2022 | Spain | Hospitalisation | Medical Records | Clinical | Non specified | Random Forest Naïve Bayes Bernoulli  Support Vector Machine Deep Learning - Neural Networks Naïve Bayes Gaussian Stochastic Gradient Descent | Random Forest 0.74 Naïve Bayes Bernoulli 0.66  Support Vector Machine 0.64 Deep Learning - Neural Networks 0.63 Naïve Bayes Gaussian 0.60 Stochastic Gradient Descent 0.52 | Clinical Decision Support and Personalized Care  Data Integration and Advanced Modelling | None |
| Prediction of Hospitalisation Cost and Length of Stay for Patients with Heart Failure Using Deep Learning^(36)^ | 2022 | Japan | Hospitalisation | Medical Records and Patient Survey | Clinical and Demographic | Non specified | Deep Learning - Deep Learning based Regression Model | AUC not available (only mean absolute error, mean squared error and coefficient of determination) | Clinical Decision Support and Personalized Care  Resource Planning and Management | None |
| Prediction of long-term hospitalisation and all-cause mortality in patients with chronic heart failure on Dutch claims data: a machine learning approach^(37)^ | 2021 | Netherlands | Hospitalisation | Medical Records | Clinical and Demographic | Until 365 days and until 1095 days (3-year) | Deep Learning - Neural Networks Elastic Net Logistic Regression Random Forest | Deep Learning - Neural Networks 0.73 and 0.73 Elastic Net 0.73 and 0.73 Logistic Regression 0.71 and 0.73 Random Forest 0.71 and 0.71 | Clinical Decision Support and Personalized Care  Resource Planning and Management | None |
| Prediction of sudden health crises owing to congestive heart failure with deep learning models^(38)^ | 2021 | United States of America | Readmission | Medical Records | Clinical and Demographic | 30-day, 60-day and 90-day | Deep Learning - Multi-Layer Perceptrons | AUC not available (only accuracy) | Clinical Decision Support and Personalized Care  Resource Planning and Management | None |
| The Price of Explainability in Machine Learning Models for 100-Day Readmission Prediction in Heart Failure: Retrospective, Comparative, Machine Learning Study^(39)^ | 2023 | Sweden | Readmission | Medical Records | Clinical, Demographic and Healthcare Utilization | 100-day | Categorical Boosting Deep Learning - Long Short-Term Memory | Categorical Boosting 0.68  Deep Learning - Long Short-Term Memory 0.67 | Clinical Decision Support and Personalized Care | None |
| Using Ensemble Machine Learning Methods for Predicting Risk of Readmission for Heart Failure^(40)^ | 2019 | United States of America | Readmission | Medical Records | Clinical | Non specified | Extra Trees Deep Learning - Neural Networks Random Forest Logistic Regression Support Vector Machine Gradient Boosting Adaptive Boosting K-Nearest Neighbors Decision Trees | Extra Trees 0.70 (95% CI: 0.69-0.71)  Deep Learning - Neural Networks 0.70 (95% CI: 0.69-0.71) Random Forest 0.69 (95% CI: 0.68-0.71) Logistic Regression 0.69 (95% CI: 0.68-0.70) Support Vector Machine 0.69 (95% CI: 0.68-0.70)  Gradient Boosting 0.67 (95% CI: 0.66-0.68) Adaptive Boosting 0.66 (95% CI: 0.65-0.67) K-Nearest Neighbors 0.65 (95% CI: 0.64-0.66) Decision Trees 0.62 (95% CI: 0.61-0.63) | Clinical Decision Support and Personalized Care  Data Integration and Advanced Modelling | None |
| Utilizing electronic health data and machine learning for the prediction of 30-day unplanned readmission or all-cause mortality in heart failure^(41)^ | 2020 | United States of America | Readmission | Medical Records | Clinical, Demographic and Healthcare Utilization | 30-day | Extreme Gradient Boosting | Extreme Gradient Boosting 0.76 | Clinical Decision Support and Personalized Care  Data Integration and Advanced Modelling  Monitoring and Real-Time Analytics  Resource Planning and Management | None |
