# Supplementary material for: Machine learning methods, applications and economic analysis to predict heart failure hospitalisation risk: a scoping review
Source: BMJ Open. 2025 Jun 25;15(6):e093495. doi: 10.1136/bmjopen-2024-093495 (PMC12198813; doi:10.1136/bmjopen-2024-093495)
Supplement: Supplementary file 3 [file bmjopen-2024-093495.reviewer_comments.pdf]

PEER REVIEW HISTORY

BMJ Open publishes all reviews undertaken for accepted manuscripts. Reviewers are asked to complete a checklist review form and are provided with free text boxes to elaborate on their assessment. These free text comments are reproduced below.

ARTICLE DETAILS

Title (Provisional)

Machine learning methods, applications, and economic analysis to predict heart failure hospitalisation risk: a scoping review

Authors

Abreu, João; Seringa, Joana; Magalhaes, Teresa

VERSION 1 - REVIEW

**Reviewer**	**1**
**Name**	**Zhang, Jinhua**
**Affiliation**	**The Affiliated Union Hospital of Fujian Medical University, Department of Pharmacy**
**Date**	**12-Oct-2024**
**COI**	**None**

This paper presents a comprehensive scoping review of machine learning models aimed at predicting heart failure hospitalization risk. It analyzes their predictive performance and economic analysis. The review is valuable for both clinical practice and healthcare policy making.

(1) This paper addresses a significant and timely topic in the healthcare sector, providing a valuable synthesis of the current landscape of ML applications in heart failure risk prediction.

(2) It systematically reviews a broad range of studies, offering a thorough overview of the predictive performance of various ML models, which is useful for both clinicians and researchers.

(3) This paper outlines clear objectives and a well-structured methodology, following established protocols for scoping reviews.

**Reviewer**	**2**
**Name**	**Marzoog, Basheer A**

Affiliation **I M Sechenov First Moscow State Medical University, World-Class Research Center "Digital biodesign and personalized healthcare"**

Date **06-May-2025**

COI **None**

The paper type is not an original article but a review paper!

Add the specificity and the sensitivity to the AUC in the results subsection in the abstract.

Supply the PRISMA checklist.

The table of the found papers needs to be clearer by using the tool “Repeat Header Rows”

The conclusion is not sound; it needs to be rewritten to suggest which type of ML is preferred according to your results.

I see 6 objectives in the introduction section, but in the abstract, you did not mention all these objectives.

In the introduction section, you need to demonstrate the currently available types of machine learning. Then discuss the used methods in heart disease, including heart failure, ischemic heart disease, lipidome, metabolome, inflammasome, different types of arrhythmia, and diastolic dysfunction.

Move the section “Strengths and limitations of this study “ after the discussion section.

Add the period of the search, from which date to which date the included search period of the included papers.

Add the language of the included papers because not all the papers are included. I still see no none English language papers included.

I don’t see a reason to repeat the published part of the review, “The protocol for this scoping review was published in the British Medical Journal in April

2024 and is available at BMJ Open (doi: 10.1136/bmjopen-2023-083188).(9)”

The flowchart is not accurately performed and lacks the reasons for the exclusion of the papers in detail.

Classify the results of the found papers in a table and include the characteristics of the groups: papers that report the first-time hospitalization and papers that report the rehospitalization.

Include in the main table of the found papers the sample size, the type of validation (internal or external), the method of model building and cross-validation, and the methods to avoid the bias of the technical challenges in studies with the small sample size.

The results section is not a place to discuss the types of machine learning.

“Approximately 41% of the articles reviewed originated from the United States of

America (U.S.A.). The dominance of research from the U.S.A. may reflect the strong research infrastructure and the availability of large healthcare datasets in the co" this is a study limitation and not need to discuss this issue in a whole paragraph.

The discussion section needs to be rewritten to discuss the obtained results and give a potential suggestion for the best ML method.

Adding a table of bias evaluation of the included studies.

Add a paragraph discussing the current challenges and future perspectives in applying ML in cardiology in clinical practice.

The papers that you included in the study must be original papers and not reviews. And they must be included in the references list.

English language improvement is obligatory for paper acceptance.

Add a figure demonstrating how gradient boosting works since it has the best performance according to the stated results.

Adding a statement on the use of the artificial intelligence at any stage in this paper.

Adding keywords to the paper after the abstract and before the introduction

Adding list of abbreviations to the paper before the list of the references

Preferred to do a meta-analysis rather than a systematic review, since you found enough studies to perform it. And make a forest tree test accordingly and give clear and exact recommendation

---

VERSION 1 - AUTHOR RESPONSE

About the points raised by Reviewer 2:

1. **Add the specificity and the sensitivity to the AUC in the results subsection in the abstract.**
   Thank you for your suggestion. As noted in the manuscript, to fulfil the third specific objective—identifying the predictive performance of ML models—we included only studies reporting comparative performance metrics, with a particular focus on AUC, as it was the most consistently reported metric across studies. Specificity and sensitivity were not uniformly available and thus could not be reliably included in the abstract results subsection. We have clarified this in the manuscript to ensure transparency regarding the data extraction criteria.
2. **Supply the PRISMA checklist.**
   Thank you for your comment. As the protocol for this scoping review was previously published and followed the PRISMA-ScR (Preferred Reporting Items for Systematic Reviews and Meta-

Analyses extension for Scoping Reviews) guidelines, we believed it was not necessary to submit the PRISMA checklist with this manuscript. However, we fully acknowledge the importance of transparent and standardised reporting. If required by the journal, we are happy to provide the completed PRISMA-ScR checklist as a supplementary file.

3. **The table of the found papers needs to be clearer by using the tool "Repeat Header Rows".**
   Thank you for your suggestion to improve the readability of the table. We have applied the "Repeat Header Rows" to *Table I – Summary of articles investigating ML approaches to predict heart failure hospitalisation risk* to enhance clarity when viewing across pages.
4. **The conclusion is not sound; it needs to be rewritten to suggest which type of ML is preferred according to your results.**
   Thank you for this suggestion. We have revised the last paragraph of the Conclusions section to clearly reflect which type of ML appears most promising based on our findings. The revised text now highlights ensemble-based models as the most effective due to their consistent performance and suitability for real-world, heterogeneous healthcare data. The updated conclusion also maintains alignment with the exploratory nature of a scoping review. The last paragraph of the *Conclusions* was changed from:
   "In conclusion, while ML offers substantial potential for improving heart failure care, further efforts are needed to expand the generalisability of models, integrate diverse data sources, and evaluate the cost-effectiveness of these technologies. Addressing these challenges will help translate the promising results of ML into widespread clinical practice, ultimately reducing hospitalisations and improving patient outcomes on a global scale." to the following revised version:
   "In conclusion, while ML offers substantial potential for improving heart failure care, ensemble-based models appear to be the most effective due to their consistent performance, capacity to manage complex and high-dimensional healthcare data, and ability to capture nonlinear interactions among clinical, demographic, and utilisation variables. Their robustness and adaptability make them especially suitable for real-world healthcare settings, where data are often heterogeneous and imbalanced. Future research should aim to expand the generalisability of these models, incorporate diverse data sources, and conduct robust economic evaluations. Addressing these challenges will help translate the promising results of ML into widespread clinical practice, ultimately reducing hospitalisations and improving patient outcomes on a global scale".
5. **I see 6 objectives in the introduction section, but in the abstract, you did not mention all these objectives.**
   Thank you for your observation. The abstract presents the main objective of the review, which is: *"To conduct a comprehensive review of existing machine learning models designed to predict hospitalisation risk in individuals with heart failure."* The six specific objectives listed

in the Introduction section were developed to guide the review process in more detail and are fully addressed throughout the Results section.

6. **In the introduction section, you need to demonstrate the currently available types of machine learning. Then discuss the used methods in heart disease, including heart failure, ischemic heart disease, lipidome, metabolome, inflammasome, different types of arrhythmias, and diastolic dysfunction.**

   Thank you for your suggestion. We acknowledge the relevance of presenting an overview of existing ML types; however, we opted not to include a detailed description of them in the Introduction, as our focus was to introduce the clinical context and main objective of the review. Given the specific aim of mapping studies on ML models predicting hospitalisation risk in heart failure, we also chose not to expand the scope to include other cardiovascular conditions or biological domains. We believe this focus allows for a clearer and more targeted contribution aligned with the purpose of a scoping review.

7. **Move the section "Strengths and limitations of this study "after the discussion section.**

   Thank you for your suggestion. As this point was also raised by the Editor, we have followed the editorial guidance and moved the "Strengths and limitations of this study" section to directly follow the abstract.

8. **Add the period of the search, from which date to which date the included search period of the included papers.**

   Thank you for your comment. The database search was conducted on March 31, 2024, without applying any date restrictions, in accordance with the published protocol. As such, all relevant studies published up to that date were considered for inclusion. We have clarified this in the Methods section to ensure transparency regarding the search period.

9. **Add the language of the included papers because not all the papers are included. I still see no non-English language papers included.**

   Thank you for your observation. As stated in the *Inclusion and Exclusion Criteria* subsection of the Methods, only studies published in English were eligible for inclusion. This language restriction was applied during the screening process, which is why no non-English language papers appear in the final selection.

10. **I don't see a reason to repeat the published part of the review, "The protocol for this scoping review was published in the British Medical Journal in April 2024 and is available at BMJ Open (doi: 10.1136/bmjopen-2023-083188).**

    Thank you for your comment. The statement referring to the published protocol appears only once in the manuscript, specifically within the *Protocol registration and information* section. As also noted by the editor, we have corrected the wording to indicate that the protocol was published in *BMJ Open*, not in the *British Medical Journal*. We believe this single mention is important to ensure transparency and traceability of the review process.

11. **The flowchart is not accurately performed and lacks the reasons for the exclusion of the papers in detail.**
Thank you for your comment. While we acknowledge the value of presenting detailed exclusion reasons in the flowchart, we have chosen to present the exclusions in a simplified manner consistent with the PRISMA-ScR guidance and with the scope of our review. Given the nature of a scoping review and the broad initial inclusion criteria, individual exclusion reasons often overlapped or were multifactorial, making detailed categorisation potentially misleading. However, the exclusion criteria are clearly described in the Methods section, and the screening process was conducted transparently. If deemed necessary by the editorial team, we would be willing to include a more detailed breakdown of exclusion reasons as a supplementary table.
12. **Classify the results of the found papers in a table and include the characteristics of the groups: papers that report the first-time hospitalization and papers that report the rehospitalization.**
Thank you for your suggestion. In our review, the included studies were already categorised according to their primary outcome—hospitalisation (first-time admissions) and rehospitalisation (readmissions). This classification is presented in the summary table. However, a detailed analysis of the specific characteristics of each patient group was beyond the scope of this scoping review, as such subgroup-level data were not consistently reported across the included studies.
13. **Include in the main table of the found papers the sample size, the type of validation (internal or external), the method of model building and cross-validation, and the methods to avoid the bias of the technical challenges in studies with the small sample size.**
Thank you for your suggestion. While we acknowledge the importance of including detailed methodological information such as sample size, type of validation (internal or external), model building approach, cross-validation method, and techniques to address potential bias in studies with small sample sizes, this level of technical detail was beyond the scope of our scoping review. Our objective was to provide a broad overview of machine learning models used to predict hospitalisation in heart failure, rather than a comprehensive methodological appraisal of each study. Nonetheless, we agree that such information could be useful in a future systematic review or meta-analysis focused specifically on methodological quality.
14. **The results section is not a place to discuss the types of machine learning.**
Thank you for your observation. Could you please clarify which part of the Results section this refers to?
15. **"Approximately 41% of the articles reviewed originated from the United States of America (U.S.A.). The dominance of research from the U.S.A. may reflect the strong research infrastructure and the availability of large healthcare datasets in the co" this is a study limitation and not need to discuss this issue in a whole paragraph.**

Thank you for your feedback. We agree that the geographical concentration of studies in the U.S.A. represents a study limitation rather than a discussion point requiring a full paragraph. We will revise this section to briefly mention it as a limitation, without overemphasising its implications in the main discussion.

16. **The discussion section needs to be rewritten to discuss the obtained results and give a potential suggestion for the best ML method.**

    Thank you for your comment. The discussion section has been revised to better reflect the obtained results of the review. Information was added to highlight the potential suggestion of ensemble-based models as the best-performing models.

17. **Adding a table of bias evaluation of the included studies.**

    Thank you for your comment. We acknowledge that assessing the risk of bias is important to appraise the quality of evidence. However, as this is a scoping review, conducting a formal bias evaluation of the included studies was beyond the scope of our objectives.

18. **Add a paragraph discussing the current challenges and future perspectives in applying ML in cardiology in clinical practice.**

    Thank you for your suggestion. While we acknowledge the importance of discussing the current challenges and future perspectives in applying machine learning in cardiology, this topic was considered beyond the scope of our scoping review, which was specifically focused on mapping existing ML models predicting hospitalisation risk in heart failure. Expanding the discussion to broader clinical implementation topics would require a separate in-depth analysis. Nonetheless, we recognise its relevance and consider it an important area for future exploration.

19. **The papers that you included in the study must be original papers and not reviews. And they must be included in the references list. English language improvement is obligatory for paper acceptance.**

    Thank you for your comment. All the papers included in this scoping review were original research articles, not reviews, and we have revised the manuscript to reference them accordingly. Additionally, the manuscript has been revised to improve the quality of the English language in line with publication requirements.

20. **Add a figure demonstrating how gradient boosting works since it has the best performance according to the stated results.**

    Thank you for your suggestion. Gradient boosting was frequently associated with strong performance in the included studies, and we agree that a visual representation could enhance understanding. However, given the heterogeneity of implementations and configurations across studies, a single figure might not accurately reflect how the method was applied in each context.

21. **Adding a statement on the use of the artificial intelligence at any stage in this paper.**

    Thank you for your comment. We believe that the use of artificial intelligence is already adequately addressed in the Introduction section, where machine learning is clearly defined as

a subset of artificial intelligence and its role in healthcare is explained. Therefore, we consider that an additional explicit statement is not necessary.

22. **Adding keywords to the paper after the abstract and before the introduction.**

    Thank you for your comment. We have added the suggested keywords after the abstract and before the introduction as requested. Keywords: Heart failure, Cardiology, Machine Learning, Hospitalization, Health Economics.

23. **Adding list of abbreviations to the paper before the list of the references.**

    Thank you for your comment. A list of abbreviations has now been added to the manuscript, placed before the list of references, as suggested**.**

24. **Preferred to do a meta-analysis rather than a systematic review, since you found enough studies to perform it. And make a forest tree test accordingly and give clear and exact recommendation.**

    Thank you for your comment. We appreciate your suggestion. However, conducting a meta-analysis and producing a forest plot falls beyond the scope of this scoping review. Our objective was to provide a comprehensive overview of the machine learning models used to predict hospitalisation risk in heart failure, including the types of models applied, data sources, and reported outcomes, rather than to quantitatively synthesize effect sizes or performance metrics. A meta-analysis would require a more homogeneous set of studies with comparable methodologies and outcome measures, which was not the case in the literature identified. Nonetheless, we agree that a future meta-analysis focusing on a specific ML model or outcome could provide additional insights.
